# Predicting spinal hypotension during cesarean delivery: what is being predicted, and when

**DOI:** 10.3389/fmed.2026.1989191

**Published:** 2026-10-12

**Authors:** Yi Zhang, Shuang Guo, Qingjun Zeng, Chaowen Fu

**Affiliations:** 1 Department of Anesthesiology, Chongqing Wanzhou Health Center for Women and Children, Chongqing, China; 2 Department of Anesthesiology, Chongqing Three Gorges Medical College Affiliated People’s Hospital, Chongqing, China

**Keywords:** cesarean delivery, clinical utility, hemodynamic monitoring, hypotension, prediction, spinal anesthesia, vasopressor

## Abstract

Spinal hypotension during cesarean delivery has generated a large prediction literature, but the term “prediction” is applied to signals obtained at very different points in the peri-spinal course. Most reviews group predictors by physiology or device. Here we organize them by when the signal becomes available and which clinical decision remains open. This changes how reported performance should be interpreted. A preoperative low-frequency/high-frequency heart rate variability threshold of 2.5, for example, showed only 35% sensitivity and discrimination around 0.60 in an independent cohort, whereas small development studies of other preoperative tools have reported areas under the receiver operating characteristic curve approaching 0.9. Pre-spinal provocation may improve discrimination while preserving time to adjust prophylaxis. Measurements obtained after intrathecal injection, and real-time algorithms operating minutes before hypotension, answer a different question because sympathetic blockade is already evolving and the decision window is narrower. Variation in hypotension definitions, treatment background, case mix, and small development samples further limits direct comparison. Routine vasopressor prophylaxis also changes what a useful predictor should do: rather than decide who receives prevention, it should help identify who needs earlier, stronger, or differently titrated treatment. Two randomized cesarean trials now support Hypotension Prediction Index-guided management, while treatment-impact evidence for true preoperative prediction remains limited.

## Introduction

Spinal anesthesia is the usual anesthetic technique for elective cesarean delivery, but hypotension after the block remains a routine clinical problem. Current consensus favors prophylactic vasopressor use and maintenance of systolic pressure near the pre-spinal baseline; the 2025 Enhanced Recovery After Surgery update again places prevention of spinal hypotension among core intraoperative measures (1, 2). Prediction is attractive for a simple reason: if vulnerability can be recognized before the block, prophylaxis can be adjusted before symptoms or fetal consequences emerge.

Yet the literature has not been addressing one uniform prediction problem. A 2021 systematic review catalogued more than 30 predictors across seven physiological and clinical domains; a later review focused on preoperative heart rate variability (HRV), and a 2026 narrative review updated Doppler, hemodynamic, pulse-oximetry, and composite approaches (3–5). Those reviews are useful maps of available technologies. They do not fully resolve a different question that becomes increasingly important as the measurement moves closer to hypotension: what information was available at that moment, and what could the anesthesiologist still change?

This Mini Review therefore organizes the evidence by timing and task. A five-minute resting recording before spinal anesthesia, a response to a postural challenge before the block, a measurement obtained one to three minutes after intrathecal injection, and an arterial-waveform alert shortly before a pressure threshold is crossed may all be described as prediction. Clinically, they are not interchangeable. They sample different physiology and leave different decision windows.

## How the evidence was reorganized

Rather than build another catalogue from scratch, we used existing reviews as anchors and checked quantitative claims against the primary reports. The pre-spinal corpus was anchored to the prospective observational studies identified by Yu et al. (3), with HRV studies cross-checked against Frandsen et al. (4). The 2026 narrative review served as a recent citation source rather than a sampling frame; a focused PubMed update and citation tracking were then carried forward through September 3, 2026 (5). Published corrections were checked when relevant. Because the anchor review excluded predictors measured after spinal anesthesia, coverage of post-spinal dynamics and real-time event detection is deliberately selective rather than exhaustive. For detailed discussion and Table 1, we retained studies when the timing of measurement, the hypotension endpoint, and the clinical decision still available at that point could be reconstructed from the primary report. Studies were especially useful when they tested transportability, compared more than one measurement window, or linked a signal to a management decision. This was a selective narrative approach rather than an exhaustive inventory or formal ranking of prediction studies.

**Table 1:** Representative studies illustrating distinct prediction tasks and clinical-utility stages.

Study	Task	n	Hypotension definition	Event rate/primary endpoint	Signal/key result	Validation/main limitation
Hanss 2005 (8)	Resting preoperative	60	Severe SBP <80 mmHg	NR	LF/HF >2.5 identified a high-risk subgroup	Within-study confirmation; historical treatment context
Bishop 2017 (9, 10)	Resting preoperative	102	SBP <90 mmHg	47.1%	Prior LF/HF 2.5 threshold: Se 35%, Sp 85%, AUC about 0.60; refit 2.0 c-statistic 0.645	Published threshold did not transport well
Yokose 2015 (11)	Resting preoperative	81	SBP <80 mmHg; symptomatic rescue also counted	63%	Only heart rate retained; AUC 0.686	Result changed with alternative hypotension definitions
Du 2022 (12)	Resting preoperative	230	10-s average SBP <80% baseline	49.1%	Multivariable AUC 0.701; LF/HF negative	Secondary analysis under active vasopressor management; unstable HRV coefficients
Kim 2021 (13)	Resting preoperative	35 analyzed	SBP <80 mmHg, <75% baseline, or symptoms	60%	FTc(W) AUC 0.922	Small single-center cohort; no independent validation
Hu 2026 (15)	Resting preoperative	84 completed	SBP <90 mmHg or >30% decrease from baseline	31.0%	Baseline LVEDV/LVESV model AUC 0.725 (95% CI 0.600–0.850)	Single-center cohort; moderate discrimination; not a standalone predictor
Dayal 2025 (16)	Resting preoperative/external validation	371	SBP <80% baseline or <90 mmHg	49.3%	PRAM AUC 0.578; modified score 0.601	Formal external validation showed weak discrimination
Özakın 2026 (17)	Resting preoperative	76	SBP <90 mmHg or ≥30% below baseline; 15 min	47.4%	SI AUC 0.757; MSI 0.747; DSI 0.693; RJV-CI 0.709; RJV-CI + SI corrected AUC 0.775	Single center; exploratory cutoffs; internal bootstrap validation only
Sakata 2017 (18)	Preoperative provocation	45	SBP decrease >=20%	78%	Postural-change LF/HF AUC 0.76	Single cohort; no independent validation
Juri 2024 (7)	Preoperative resting + provocation	40	Post-spinal hypotension until delivery	65%	Baseline FTc 0.591; position-induced change 0.882	Improvement came from provocation, not temporal proximity
Hao 2026 (19)	Preoperative provocation	54	SAIH within 15 min after neuraxial block	63%	Position-induced carotid FTc change AUC 0.872; Se 94.1%, Sp 75.0%	Single-center cohort; external validation pending
Kuwata 2018 (6)	Preoperative + post-spinal	50	SBP <90 mmHg or <80% baseline	64%	PVI AUC 0.751 pre-spinal; 0.793 at 1 min post-spinal	Strongest signal already samples response to the block
Jendoubi 2021 (20)	Preoperative + post-spinal	100	SBP decrease >=20% or SBP <100 mmHg	80%	Baseline ANI non-discriminatory; post-spinal AUC 0.602–0.630	Weak discrimination; limited lead-time interpretation
Frassanito 2022 (22)	Real-time impending-event	48 analyzed	MAP <65 mmHg for >=1 min	47.9% patient-level	HPI AUC 0.913 at 3 min	Retrospective repeated-event analysis; same pressure waveform informed index and endpoint
Shih 2026 (29)	Real-time + clinical utility	171	MAP <65 mmHg	Primary endpoint TWA hypotension	HPI-guided group TWA 0.08 mmHg vs. 0.30 and 0.89 mmHg controls	Randomized utility evidence; task differs from preoperative susceptibility
Abd Halim 2026 (30)	Real-time + clinical utility	96 analyzed	MAP <65 mmHg	Primary endpoint TWA hypotension	HPI-guided TWA 0.24 [0, 0.70] vs. 1.67 [0.71, 2.57] mmHg control	Second obstetric RCT; alarm 85 with additional assessment at 50–84

ANI, Analgesia Nociception Index; AUC, area under the receiver operating characteristic curve; FTc, corrected carotid flow time; HPI, Hypotension Prediction Index; HRV, heart rate variability; LF/HF, low-frequency/high-frequency ratio; LVEDV, left ventricular end-diastolic volume; LVESV, left ventricular end-systolic volume; MAP, mean arterial pressure; PVI, pleth variability index; PRAM, pulse rate-age-mean arterial pressure score; SAIH, spinal anesthesia-induced hypotension; SBP, systolic blood pressure; Se, sensitivity; Sp, specificity; TWA, time-weighted average; NR, not reported; DSI, diastolic shock index; MSI, modified shock index; RJV-CI, right internal jugular vein collapsibility index; SI, shock index.

We classify measurements and clinical tasks, not whole papers. Some studies naturally cross the boundaries. Kuwata et al., for example, reported pleth variability index (PVI) both before and after spinal anesthesia, while Juri et al. compared resting corrected carotid flow time (FTc) with the change produced by a preoperative head-down tilt (6, 7). That overlap is informative because the same physiological variable can answer a different clinical question when sampled at another point in the peri-spinal sequence. Figure 1 summarizes the framework; Table 1 provides representative studies and the corresponding decision window.

**Figure 1:**
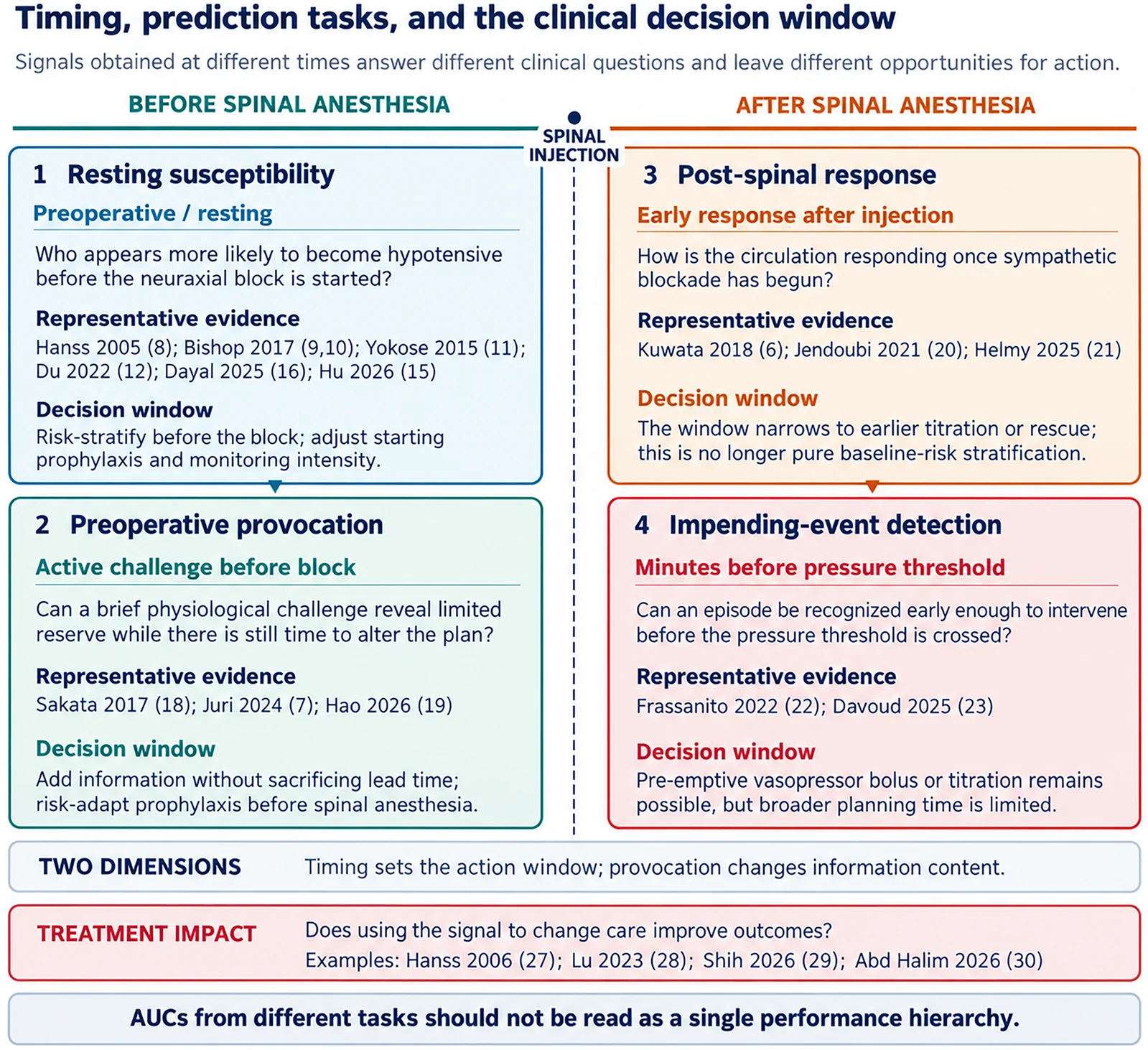
Timing, prediction tasks, and the clinical decision window. Resting measurements and preoperative provocation are available before spinal anesthesia and retain a broader window for risk-adapted prophylaxis. Measurements obtained after intrathecal injection can already contain part of the response to sympathetic blockade, while short-horizon algorithms move closer to impending-event detection. Treatment impact is shown as a separate evaluation layer because it asks whether acting on a signal improves care. Timing and physiological provocation are related but nonidentical dimensions, and AUCs across these tasks should not be interpreted as a single performance hierarchy. AUC, area under the receiver operating characteristic curve.

## One label, several prediction tasks

The most literal form of preoperative prediction is a resting measurement obtained before neuraxial anesthesia, without deliberately perturbing the circulation. The intended output is baseline susceptibility. Early HRV work shows both the appeal and the difficulty. Hanss et al. reported that a high pre-spinal low-frequency/high-frequency (LF/HF) ratio identified women at greater risk of severe hypotension (8). Bishop et al. later tested the proposed LF/HF threshold of 2.5 in an independent cohort. Sensitivity fell to 35%, specificity was 85%, and discrimination was about 0.60. A lower threshold of 2.0 fitted in the new cohort improved little, with a c-statistic of 0.645 (9, 10). That is a transportability problem, not a minor adjustment of cutoff.

Results across resting markers have been uneven. Yokose et al. found that only heart rate remained independently predictive, with an area under the receiver operating characteristic curve (AUC) of 0.686; baseline PVI, perfusion index, LF/HF, and entropy were not retained (11). In Du et al.'s 230-patient analysis, a multivariable HRV and blood-pressure model performed similarly (AUC 0.701), LF/HF again was not associated with the endpoint, and several model estimates were unstable after adjustment (12). Smaller development cohorts have produced much higher estimates. Carotid FTc in 35 analyzed patients reached AUCs of 0.905 and 0.922, while a 45-patient arterial-stiffness neural-network model reported 0.89; neither had independent validation (13, 14). More recent tests have been less dramatic. Baseline echocardiographic ventricular volumes yielded an AUC of 0.725 among 84 completers, whereas external validation of the pulse rate-age-mean arterial pressure (PRAM) score in 371 women produced 0.578; adding early post-spinal tachycardia raised it only to 0.601 (15, 16). A high AUC in a small development sample and weak performance after a rule travels are not equivalent evidence.

A recent prospective study also examined simple bedside measures available before intrathecal injection. Özakın et al. analyzed 76 women and reported AUCs of 0.757 for shock index (SI), 0.747 for modified shock index (MSI), 0.693 for diastolic shock index (DSI), and 0.709 for the right internal jugular vein collapsibility index (RJV-CI). A model combining RJV-CI with SI had an optimism-corrected AUC of 0.775 (17). These measures fit the resting-susceptibility window well because they are obtainable before neuraxial perturbation and require little more than routine vital signs or a brief bedside ultrasound. The proposed cutoffs, however, were derived in the same single-center cohort and were explicitly treated as exploratory.

Provocation asks a different question. The neuraxis is still untouched, but the circulation is deliberately challenged. Sakata et al. used postural change on the day before cesarean delivery and reported an HRV-based AUC of 0.76 (18). Juri et al. provide a particularly useful within-study contrast: resting FTc discriminated poorly (AUC 0.591), whereas the change induced by a 15-degree Trendelenburg position before spinal anesthesia reached 0.882 (7). Hao et al. later used a supine-to-left-lateral positional change before neuraxial block; the change in carotid FTc reached an AUC of 0.872 in 54 women, with 94.1% sensitivity and 75.0% specificity at a 6.49-ms cutoff (19). The gain in these studies did not come from waiting until hypotension was closer. It came from asking the cardiovascular system to reveal reserve while there was still time to alter the pre-spinal plan.

After intrathecal drug has been given, the prediction task changes again because the measurement can contain part of the response to sympathetic blockade. Kuwata et al. reported AUCs of 0.751 for pre-spinal PVI and 0.793 one minute after spinal anesthesia; only the post-spinal value remained independently associated with hypotension (6). Jendoubi et al. found no useful separation with baseline Analgesia Nociception Index, whereas the three-minute post-spinal change showed weak discrimination, with AUCs of 0.602 to 0.630 and specificity of only 34% to 36% (20). A 2025 femoral-Doppler study performed under prophylactic norepinephrine reported AUCs of 0.99 and 0.96 for post-spinal changes in pulsatility and resistive indices (21). Values that close to perfect do not mean baseline susceptibility has suddenly become easy to predict. They may partly reflect a hemodynamic response already in progress. The same timing effect appears with the Hypotension Prediction Index (HPI): discrimination rose from an AUC of 0.913 three minutes before hypotension to 1.00 one minute before the event (22). As lead time shortens, risk estimation starts to become impending-event detection.

Real-time algorithms sit even closer to the event. Frassanito et al. evaluated HPI from a noninvasive arterial waveform and reported an AUC of 0.913 three minutes before mean arterial pressure (MAP)-defined hypotension (22). The analysis was retrospective, included repeated events, and derived both the index and the MAP endpoint from the same evolving pressure waveform. Davoud et al. instead modeled MAP directly from previous MAP, phenylephrine exposure, and intrathecal bupivacaine. Their autoregressive model achieved a one-minute root-mean-square error of 3.6 mmHg in development/testing and 4.2 mmHg in an independent 84-patient validation dataset (23). These may be useful short-horizon tools. They should not be read as evidence that a better baseline susceptibility marker has been found.

## Why performance does not travel cleanly

Before comparing predictors, the endpoint itself deserves attention. Klöhr et al. identified 15 definitions of spinal hypotension in 63 studies; when those definitions were applied to the same prospective cohort, the incidence ranged from 7.4% to 74.1% (24). Table 1 shows the same issue in contemporary work, where absolute pressure thresholds, relative decreases, and treatment-linked definitions coexist. Event prevalence does not mathematically determine AUC, but the endpoint, treatment background, and case spectrum all change what a discrimination estimate means and how well it travels.

Yokose et al. provide a rare direct demonstration. When hypotension was redefined as a systolic blood pressure decrease of at least 20%, 67 of 81 women met the endpoint and none of the pre-anesthetic parameters differed between groups. With a decrease of at least 30%, 50 of 81 met the endpoint and heart rate again became the only differing parameter (11). The patients and predictors did not change; the conclusion changed when the outcome definition did.

Small development samples add another layer of uncertainty, especially when they are paired with data-driven threshold selection or multivariable modeling. Current prediction-model guidance emphasizes adequate sample size, control of optimism, clear separation of development from evaluation, and calibration as well as discrimination (25, 26). Bishop evaluated a published LF/HF threshold using sensitivity, specificity, and c-statistic, while Dayal's external validation of PRAM centered on ROC discrimination and cutpoints (9, 16). Those metrics are useful, but they do not answer every clinical question. Discrimination, calibration, and clinical utility are distinct; an AUC cannot establish that predicted risks are well calibrated or that acting on a chosen threshold yields net benefit.

## The clinical question has moved

If prevention were reserved for a high-risk subgroup and everyone else simply observed, the role of prediction would be straightforward. Elective cesarean anesthesia is no longer practiced that way. Consensus guidance recommends routine prophylactic vasopressors, and enhanced-recovery guidance treats prevention of spinal hypotension as a standard intraoperative measure (1, 2). Instability nevertheless persists. In Du et al.'s 230-patient cohort, vasopressors were actively used to keep systolic pressure within 10% of baseline, yet 49.1% of women still crossed the study's hypotension threshold (12). Residual events under active management make the contemporary prediction problem more, not less, clinically relevant.

Treatment background is another reason that prediction estimates do not travel cleanly. A cohort managed with routine phenylephrine or norepinephrine prophylaxis is not observing the same event process as a cohort in which vasopressors are given only after pressure falls. Prophylaxis can change both the frequency and the timing of recorded hypotension and may attenuate, or sometimes reshape, the association between a baseline marker and the endpoint. An AUC estimated under one vasopressor protocol should therefore not be assumed to transport unchanged to another setting (1, 2, 12).

The useful role of a preoperative marker is therefore narrower than simply deciding who receives prevention. A credible marker might support a higher starting vasopressor dose, shorter measurement intervals, earlier escalation, a different drug strategy, or more intensive hemodynamic monitoring. Few preoperative prediction studies have actually been designed around those choices.

Only a small number of studies have asked what happens when a signal changes care. Hanss et al. used LF/HF to identify a high-risk subgroup and then tested prophylactic strategies; severe hypotension occurred in 20 of 23 high-LF/HF controls, whereas prophylactic vasopressor treatment prevented hypotension in 19 of 20 high-risk patients (27). Nearly two decades later, Lu et al. tested ultrasound-guided fluid management. Pre-spinal fluid exposure fell substantially, but hypotension did not, occurring in 65% and 60% of the two groups (28). Real-time HPI management has now entered this treatment-impact space more convincingly. In Shih et al., 171 randomized patients had a median time-weighted average (TWA) hypotension of 0.08 mmHg with HPI-guided care versus 0.30 and 0.89 mmHg with continuous and oscillometric monitoring (29). Abd Halim et al. analyzed 96 women and likewise found a lower TWA of MAP below 65 mmHg with HPI-guided management, 0.24 [0, 0.70] versus 1.67 [0.71, 2.57] mmHg (30). Shih has also entered a 2026 meta-analysis of HPI randomized trials across surgical populations (31). Clinical utility is no longer supported by a single obstetric experiment. Those trials show benefit of the monitoring-and-treatment strategy that was tested; they do not, by themselves, provide a new estimate of HPI discrimination or validate an earlier ROC-derived threshold.

## A trial that would answer the current question

The next preoperative study does not need another retrospectively optimized threshold. It needs to randomize the decision strategy. A prespecified provocation marker could be measured in all women before spinal anesthesia, followed by randomization to marker-guided prophylaxis or usual care. Routine prophylaxis would remain in both arms. In the guided arm, a prespecified high-risk result would intensify the starting vasopressor strategy, while a low-risk result would leave standard prophylaxis unchanged. The marker would remain concealed in usual care. This design tests whether using the marker improves management, rather than whether stronger prophylaxis helps a group already selected as high risk. Hanss et al. provide an early precedent: all 80 women were randomized to control or LF/HF-guided treatment, with targeted prophylaxis assigned according to baseline LF/HF in the treatment arm (27).

Several choices would need to be fixed before enrollment. The primary endpoint should not vary with the predictor result; a time-weighted hypotension measure or a clearly prespecified systolic threshold aligned with consensus targets would be preferable to a *post hoc* composite. Vasopressor exposure, reactive hypertension, bradycardia, maternal nausea and vomiting, and neonatal acid-base outcomes should sit beside the blood-pressure endpoint. The available provocation studies are still single-cohort evaluations without independent external validation (7, 18, 19), so the marker should be measured in both arms and the threshold frozen in advance. Concealment in usual care would provide a prospective test of transportability under contemporary prophylaxis, while disclosure in the guided arm would test whether acting on the information improves care. Du et al.'s 49% residual event rate offers a rough planning reference, but a definitive trial should use a contemporary local control rate rather than import a historical incidence mechanically (12).

## Discussion

It is tempting to read this literature as a contest between physiological signals. That framing misses the main clinical distinction. A resting preoperative measurement, a challenge applied before the block, a response recorded after sympathetic blockade has started, and a real-time alert immediately before hypotension are different tasks. Their AUCs do not belong in a single league table.

Recent progress looks different once timing is kept visible. Provocation studies suggest that discrimination can improve before spinal anesthesia without sacrificing lead time. Post-spinal and real-time approaches can draw on physiology that is already evolving, which may be clinically useful but does not solve the baseline-risk problem. HPI also illustrates why a threshold cannot be separated from the way it is used. Frassanito's performance study identified an empirical cutpoint of 30 three minutes before hypotension, whereas the Shih randomized protocol used 85 as the treatment trigger; the broader randomized-trial meta-analysis likewise records 85 for that trial (22, 29, 31). Success of the management protocol validates the trigger and treatment strategy that were tested, not the earlier ROC-derived cutpoint.

The clearest gap remains before spinal anesthesia. Preoperative markers need independent evaluation, prespecified thresholds, and treatment-impact trials conducted on top of modern prophylaxis. The relevant question is no longer only “Can this variable discriminate?” It is “Does acting on it improve blood-pressure control or maternal symptoms without excessive vasopressor exposure?” Real-time HPI management, meanwhile, has moved beyond an isolated proof of concept: at least two randomized cesarean trials report lower hypotension burden, and the wider randomized literature has already been synthesized quantitatively (29–31). Clinical-utility evidence is accumulating for real-time detection; true preoperative prediction remains much less mature.

## Limitations

This framework has several limitations. The review was not systematic and did not include a formal risk-of-bias assessment. Its structured anchor corpus is strongest for pre-spinal predictors because the 2021 systematic review excluded measurements obtained after spinal anesthesia; post-spinal dynamic studies and real-time prediction were added through a focused PubMed update and citation tracking, so those later tasks are selectively sampled rather than exhaustive. The update was limited to literature identifiable in PubMed and the selected source reviews, predominantly in English. The framework is also interpretive. Although it is drawn chronologically, it is not purely temporal: timing determines the clinical decision window, whereas physiological provocation can change the information content of a measurement. The two dimensions overlap, but they are not the same.

## Conclusion

In cesarean spinal anesthesia, “prediction” currently covers several distinct tasks. Timing affects both the physiology being sampled and the action that remains available. Resting susceptibility, preoperative provocation, post-spinal response, and impending-event detection should not be interpreted as interchangeable performance problems. Real-time HPI management now has randomized obstetric support from more than one trial; true preoperative prediction remains less mature in transportability and treatment impact. The next useful study is not another internally optimized cutoff. It is a prospective strategy trial in which a prespecified pre-spinal signal is measured in all women, concealed under usual care, and used to guide prophylaxis only in the marker-guided arm.
